# PtSe_2_-Based Heterostructures on GaP Nanowires: Interface Formation and Photoelectrical Properties

**DOI:** 10.3390/nano16181161

**Published:** 2026-09-15

**Authors:** Agáta Laurenčíková, Michaela Sojková, Ivana Lettrichová, Stanislav Hasenöhrl, Peter Eliáš, Dušan Pudiš, Jaroslav Kováč, Martin Hulman, Jaroslav Kováč, Alica Rosová, Peter Švec, Igor Píš, Jozef Novák

**Affiliations:** 1 Institute of Electrical Engineering, Slovak Academy of Sciences, SK-841 04 Bratislava, Slovakia; michaela.sojkova@savba.sk (M.S.); alica.rosova@savba.sk (A.R.); igor.pis@savba.sk (I.P.);; 2 Department of Physics, University of Žilina, SK-010 26 Žilina, Slovakia; ivana.lettrichova@uniza.sk (I.L.);; 3 Institute of Electronics and Photonics, Slovak University of Technology, SK-812 19 Bratislava, Slovakia; 4 Institute of Physics, Slovak Academy of Sciences, SK-845 11 Bratislava, Slovakia

**Keywords:** GaP nanowires, PtSe_2_, Ga_2_O_3_, heterostructure, optoelectronic device, interface formation

## Abstract

PtSe_2_/GaP nanowire heterostructures were fabricated and characterized to explore their optoelectronic response under broadband illumination. Few-layer PtSe_2_ was synthesized on GaP substrates with and without nanowires by selenization of a pre-deposited Pt layer. Such PtSe_2_/III-V heterostructures are attractive for next-generation photodetectors because they combine the tunable band structure and environmental stability of PtSe_2_ with the wide bandgap and established epitaxial technology of GaP. Structural and spectroscopic characterization confirms the formation of PtSe_2_ and reveals pronounced interfacial reactions between GaP and the chalcogen/metal layers. Cross-sectional STEM and compositional analysis show a heterogeneous interface comprising a PtSe_2_-rich top region containing embedded Ga_2_Se_3_ crystallites and an underlying predominantly amorphous Ga_2_O_3_-rich interlayer on GaP. Optical measurements reveal absorption edges in the 1.4–1.7 eV range attributed to PtSe_2_ and a higher-energy edge around 2.6 eV associated with GaP. Two-terminal current–voltage measurements in the dark and under illumination exhibit rectifying, photodiode-like behavior with a broadband photoresponse arising from both GaP and PtSe_2_. These results demonstrate that PtSe_2_-rich/Ga_2_O_3_-rich/GaP heterostructures containing Ga_2_Se_3_ crystallites obtained by selenization of thin Pt layers on GaP provide a simple route to photoactive interfaces combining two-dimensional chalcogenides with III-V semiconductors.

## 1. Introduction

Two-dimensional (2D) materials have become one of the fastest-growing platforms for next-generation nanoelectronics, photonics, and chemical sensing technologies over the past decade. A key feature of 2D structures is the absence of dangling surface bonds, which results in chemically smooth interfaces. This enables the vertical stacking of different 2D materials into heterostructures (HSs) [1]. While graphene initially spurred the expansion of 2D material research, its absence of an intrinsic bandgap limits its use in field-effect transistors and optoelectronic devices. This has led to extensive research into alternative layered materials with semiconducting or semimetallic properties.

Transition metal dichalcogenides (TMDCs) are among the most-studied groups of 2D materials. The general chemical formula is MX_2_, where M is a transition metal, and X is a chalcogen (S, Se, or Te). Their crystal structure features covalently bonded layers held together by weak van der Waals forces, allowing straightforward exfoliation to a monolayer [2]. Several TMDCs have tunable band gaps ranging from 1 to 2 eV, transitioning from indirect in bulk form to direct in monolayer form, making them highly promising for electronics, optoelectronics, and catalysis [3]. Electronic properties of TMDC materials include semiconducting, metallic, and even superconducting states characterized by band structures that can be readily modified by varying the number of layers, applying external strain, or introducing defects. Due to their wide tunability, TMDCs are actively studied for next-generation sensors, energy storage, solar cells, photodetectors, field-effect transistors, solar-driven hydrogen production, and biotechnology [4].

Among group-10 TMDCs, platinum diselenide (PtSe_2_) has recently garnered significant interest as a highly promising material due to its excellent environmental stability and advantageous optoelectronic, electrical, and catalytic properties. These qualities make PtSe_2_ a competitive candidate for advanced photodetection, energy-harvesting technologies, and next-generation solar cells [5]. A key feature of PtSe_2_ is its distinct atomic-layer-dependent electronic structure. The monolayer PtSe_2_ functions as an indirect semiconductor with a band gap of approximately 1.2 eV [6,7]. At the same time, the bulk material becomes a semimetal with nearly no band gap. Even a few extra layers tend to introduce metallic-like behavior. Strong interlayer coupling, especially in chalcogen-based layered materials containing selenium, can significantly modify the electronic band structure. This coupling can cause significant band-gap narrowing or even complete gap closure, as seen in studies of two-dimensional materials such as InSe and PtSe_2_ [8]. In addition to thickness effects, point defects, such as selenium vacancies, can alter the local bonding environment and induce transitions in electronic properties [9].

Realizing the full technological potential of PtSe_2_ requires controlled, large-area synthesis on low-cost and optically transparent substrates. Thermally assisted conversion (TAC) methods have become reliable for wafer-scale growth of PtSe_2_. TAC allows direct selenization of pre-deposited Pt films, providing precise control over lateral size and thickness by adjusting the initial Pt layer thickness and the substrate size. In 2015, Wang et al. were the first to produce a single-crystal monolayer of PtSe_2_ through direct selenization of a Pt(111) substrate [10]. Most reported PtSe_2_ layers have been formed by selenizing pre-deposited Pt thin films using the TAC method [11,12]. However, producing ultrathin PtSe_2_ below the critical thickness at which the semiconductor-to-semimetal transition occurs remains a major challenge.

The growth and properties of PtSe_2_ are heavily dependent on the substrate used, thus the choice further influences film quality and cost. Variations in grain size, crystallographic texture, strain, and interfacial chemistry are also inherently affected. Common substrate materials include Si wafers [13], Si/SiO_2_ [14], and sapphire [15]. Developing a reliable method for directly fabricating high-quality, large-area monolayer or few-layer films on cost-effective substrates [16] is essential.

Beyond traditional substrates, 2D PtSe_2_ films have also been successfully grown on gallium nitride [17,18], demonstrating that PtSe_2_ can be integrated with III-V semiconductor surfaces. However, detailed studies of PtSe_2_ growth on III-V substrates are limited. This is important because III-V materials differ significantly in surface structure, lattice parameters, and chemical termination, all of which are crucial for film growth, continuity, and crystalline quality. PtSe_2_ is a layered material with tunable electronic properties, while GaP is a wide-bandgap semiconductor known for its strong optical absorption and electronic stability. Combining these two materials into a van der Waals heterostructure enables the integration of their complementary properties, potentially improving device performance. Parmar et al. performed density functional theory (DFT) calculations to examine the structural, electronic, and optical properties of the PtSe_2_/GaP heterostructure and to assess its potential for nanoelectronics and optoelectronics applications [19].

While TAC allows for the synthesis of large-area PtSe_2_ films, it cannot produce monolayers; CVD is preferred for growing monolayers. It has been shown that atomic-scale substrate edges can significantly enhance the growth of certain TMDC materials. Therefore, the realization of controlled-edge epitaxy of TMDCs opens the door to the construction of additional monolayer components for future monolayer electronics [20]. Spatially connected TMDC lateral heterojunctions are essential for monolayer electronic and optoelectronic devices. Direct growth of lateral heterojunctions is challenging because TMDC alloys are thermodynamically favored. The two-step epitaxial growth of lateral WSe_2_/MoS_2_ heterojunctions demonstrates that WSe_2_ edges can drive MoS_2_ growth despite a significant lattice mismatch, enabling the controlled formation of heterojunctions with atomically sharp interfaces [21]. An edge-rich MoS_2_ nanoarray grown on edge-oriented 3D graphene was fabricated via chemical vapor deposition to maximize exposure of photocatalytically active sites. The geometrically designed 3D graphene provides a large surface area, enhanced light absorption, and optimized charge transfer between graphene and MoS_2_, thereby improving optical and electrical properties [22]. The microstructure evolution of MoS_2_ thin films strongly depends on growth conditions and film thickness, transitioning from amorphous to microcrystalline. Microstructural optimization, similar to edge-rich or orientation-controlled growth, effectively reduces recombination at the MoS_2_/p-Si interface and significantly boosts photovoltaic performance [23]. Gallium phosphide (GaP) is a promising platform due to its high thermal stability and well-defined surface chemistry, both of which are favorable for systematic studies of surfaces and interfaces. Additionally, GaP can be prepared as nanowires or nanocones. GaP nanocones are typically fabricated on GaP(111) substrates [24]. This crystallographic orientation allows for the formation of nanocones with a hexagonal base and six triangular sidewalls, each derived from well-defined crystallographic planes. The sidewalls contain a high density of nanometer-scale steps, resulting in a tapered surface with numerous nanoedges. These nanoedges can serve as preferential sites for edge-controlled growth of TMDC materials [25]. However, the interaction between PtSe_2_ and GaP, including strain effects from lattice and thermal mismatch, nucleation behavior, growth mode, and potential modifications of crystallinity, texture, or in-plane orientation, remains largely unexplored. A thorough understanding of these surface and interface effects is crucial for the controlled growth of high-quality PtSe_2_ films and for evaluating the potential of PtSe_2_/GaP heterostructures for future electronic and optoelectronic applications.

In this study, we report the growth of few-layer PtSe_2_ films on three types of substrates: (i) planar GaP, (ii) GaP covered with nanostructures, and (iii) semi-insulating sapphire, through selenization of pre-deposited platinum layers. The PtSe_2_ formation on these substrates is confirmed by Raman and XPS spectroscopy and optical measurements, which also reveal the presence of Ga-Se related phases on GaP.

The integration of PtSe_2_ with GaP is, however, strongly influenced by the interfacial chemistry of the GaP surface. GaP surfaces exposed to ambient conditions are known to develop native oxide layers or adsorb oxygen species before further processing. Under high-temperature treatment, these species can react to form gallium oxide (Ga_2_O_3_) in the near-surface region. In our case, Pt was sputtered onto GaP substrates that likely already contained native or oxygen-adsorbed layers, and subsequent selenization at 550 °C provided sufficient thermal energy for oxygen to diffuse and react in the near-surface region [26,27].

Cross-sectional TEM, STEM-EDS, HAADF-STEM, and XPS analyses show that the interface is heterogeneous. A PtSe_2_-rich upper region contains Ga_2_Se_3_ crystallites, whereas the region adjacent to GaP is predominantly amorphous and Ga_2_O_3_-rich. Accordingly, the structure is described as a PtSe_2_-rich/Ga_2_O_3_-rich/GaP heterostructure containing Ga_2_Se_3_ crystallites.

In the following sections, we demonstrate the growth of PtSe_2_ films on both planar and nanostructured GaP substrates via selenization of thin Pt layers. We further show that the resulting heterostructures are characterized by a PtSe_2_-rich/Ga_2_O_3_-rich/GaP heterostructure with embedded Ga_2_Se_3_ crystallites, and investigate their structural, optical, and electrical properties to assess their potential as photoactive interfaces for PtSe_2_-based optoelectronic devices.

## 2. Experimental Methods

Gallium phosphide nanowires (NWs) were grown on a p-type Zn-doped (*p* = 1 × 10^18^ cm^−3^) GaP(111)B substrate using a combination of standard 2D growth and vapor–liquid–solid (VLS) technique with 30 nm gold particles as seeds. These seeds formed from a 0.5 nm thick Au layer during the initial stage of NW growth. The process was carried out in an AIX 200 MOVPE low-pressure reactor at 650 °C and 100 mbar. The NWs were doped with Zn to achieve p-type conductivity. They grew at approximately 120 nm/min. Depending on the growth duration, nanowires with heights between 1.5 and 5.5 µm can be obtained; the nanowire array used in this study had a height of approximately 1.5–1.8 µm.

PtSe_2_ few-layer films were grown by a two-step method on top of three types of substrates—a flat GaP substrate, nanostructured GaP substrate, and c-plane sapphire substrate. At first, DC magnetron sputtering in an Ar atmosphere (10^−3^ mbar) at room temperature from a platinum target was used to fabricate Pt films. The DC power and emission current were set to 580 W and 0.18 A, respectively. The pre-deposited Pt layers (3 nm thick) were then selenized in a custom-designed CVD chamber [12]. In particular, the Pt layer was annealed in selenium vapor at 550 °C in an N_2_ atmosphere at ambient pressure. The substrate, together with the Se powder, was placed in the center of the furnace, ensuring that the substrate and the powder had the same temperature during growth. The annealing time and heating rate were 30 min and 25 °C/min, respectively.

X-ray photoelectron spectroscopy (XPS) was employed to investigate the chemical composition and bonding states in PtSe_2_ films grown on nanostructured and planar GaP substrates. The measurements were performed using a Thermo Scientific Nexsa G2 Surface Analysis System (Thermo Fisher Scientific, UK) equipped with a monochromatized Al K_α_ X-ray source (hν = 1486.68 eV) and a hemispherical analyzer. The spectra were acquired in constant energy mode at a pass energy of 50 eV, with a total energy resolution of approximately 0.6 eV, at take-off angles of 90°, corresponding to information depth of approximately ~7 nm. The sample with vertically aligned GaP NWs was measured at a take-off angle of 25° to reduce the contribution of signals from the exposed substrate between the nanowires. A dual-beam charge compensation system was employed during the measurements to prevent any localized charge build-up. The binding energy scale for the heterostructure samples was referenced to the Pt 4f_7/2_ peak of PtSe_2_ (73.01 ± 0.06 eV) [12], whereas binding energies for the GaP reference sample calibrated using the adventitious carbon C 1s peak at 284.8 eV. Survey spectra for both types of both samples heterostructures confirmed the presence of Ga, P, Pt, Se, O and adventitious carbon, consistent with PtSe_2_/GaP heterostructures covered by containing an interfacial gallium-oxide layer and a thin surface carbon contamination layer. The narrow-range XPS spectra were fitted with the open-source KherveFitting software (version 1.80) [28] using Voigt line shapes and a Shirley background. Relative atomic concentrations were determined from peak areas after correction using the corresponding relative sensitivity factors provided in the Avantage software package (version 6.10.0).

For transmission electron microscopy (TEM) analysis a layer deposited on flat GaP substrate was chosen and a cross-section TEM specimen was prepared using the focused ion beam (FIB) technique. Ga ions were used for the sample milling and thinning. Before TEM specimen preparation an amorphous carbon was deposited ex situ to protect sample surface from Ga ion beam destruction. Further protecting layers were deposited onto zone of interest just before FIB treatment in the dual beam equipment. First a thin Pt layer deposited using electron beam and after a thicker Pt layer using Ga ions.

Conventional JEOL JEM1200EX (JEOL Ltd., Akishima, Tokyo, Japan) operated at 120 kV and a probe-corrected FEI/TFS Titan Themis 300 electron microscope (FEI Company, Hillsboro, OR, USA) operated in scanning mode (STEM) at 200 kV accelerating voltage were used. For STEM and STEM-EDS experiments the probe convergence angle was set to 17.5 mrad, the presented micrographs were acquired using a high-angle annular dark field (HAADF) detector with calibrated collection angles 136–200 mrad and an annular bright field (ABF) detector with calibrated collection angles 12–24 mrad. The microscope was equipped with ChemiSTEM energy-dispersive spectroscopy (EDS) technology. EDS data were acquired by serial scanning across a defined area of the specimen with cumulative recording of EDS spectra at each probe position.

Heterojunction devices were fabricated by evaporating indium contacts on the entire backside surface of the p-GaP substrate and on localized circular areas (200 μm diameter) through a metallic mask on top of PtSe_2_. Both the indium layers were 60 nm thick. The contacts were annealed at 450 °C in a forming gas atmosphere. Room-temperature current–voltage (*I*–*V*) characteristics of the prepared devices were measured using an Agilent 4155C semiconductor parameter analyzer in the dark and under focused halogen white light.

The optical bandgap was determined using Tauc’s formula. The absorbance (*A*) of the sample was derived from reflectance (*R*) and transmittance (*T*) measurements according to the conservation equation:

*A* = 1 − *T* − *R* (1)

**Figure 1:**
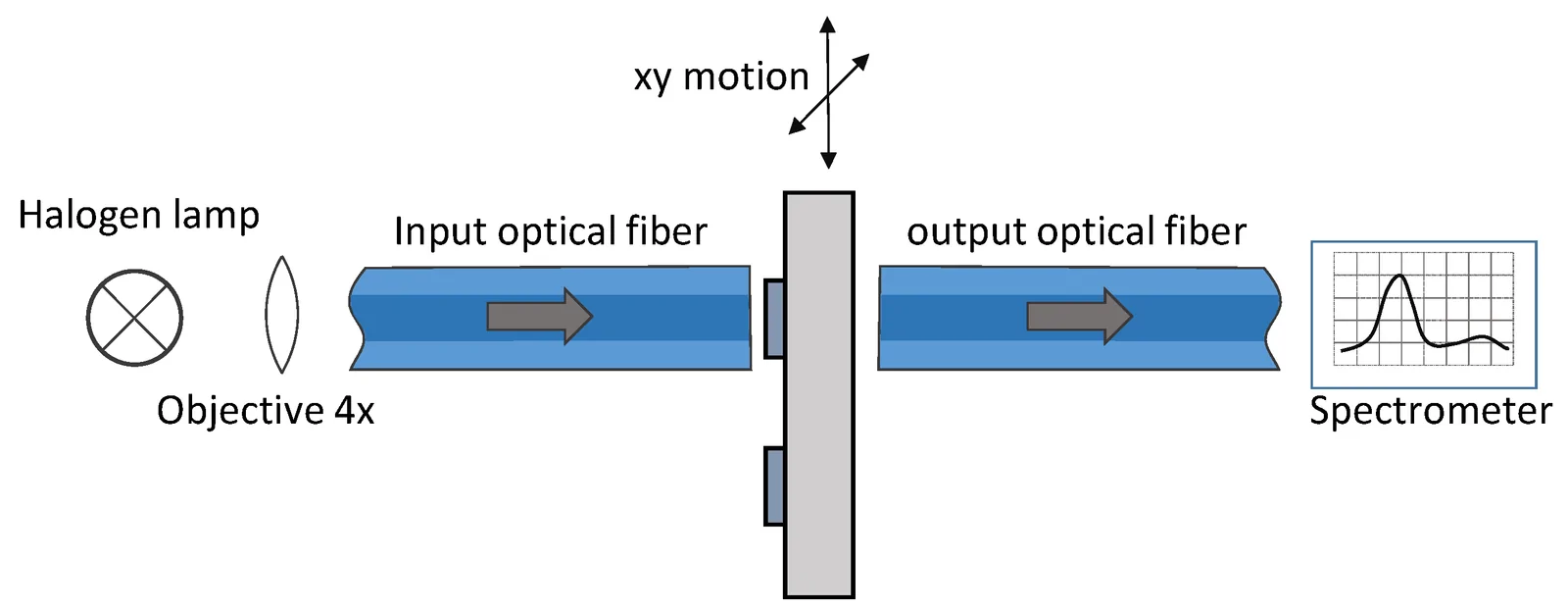
Experimental set-up for transmission measurements.

For the spectral transmission measurements of the prepared structures, the sample surface was vertically illuminated by a halogen lamp coupled to an optical fiber with a 50 µm core diameter. The transmitted light was coupled into the output optical fiber and spectrally analyzed by the OceanOptics HR2000+ spectrometer (Ocean Optics, Inc., Dunedin, FL, USA) in the range of 350–1050 nm. The experimental setup for transmission measurements is shown in Figure 1 for clarity.

**Figure 2:**
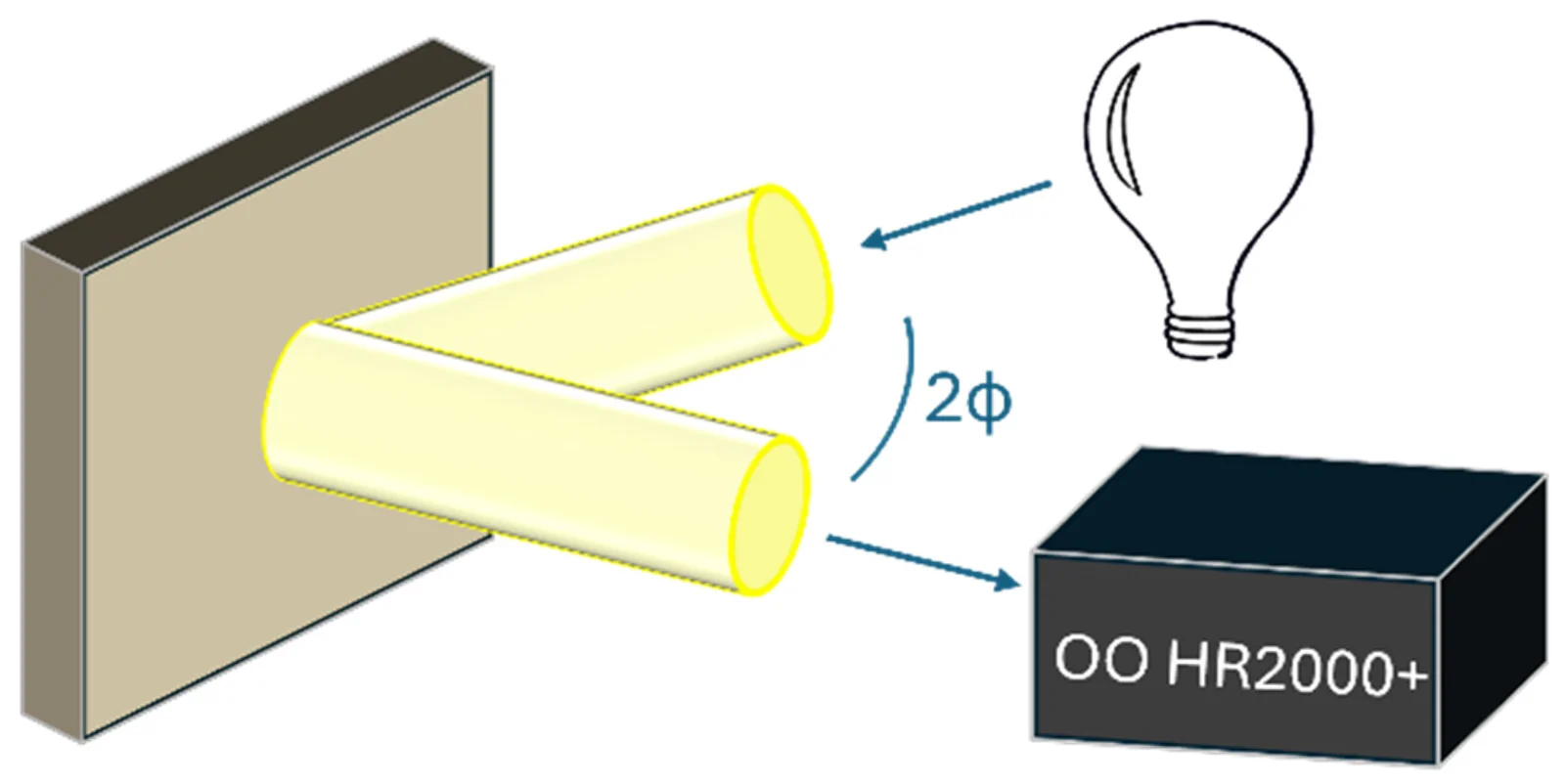
Experimental set-up for reflection measurements.

The reflection measurements were conducted using a rotational stage to determine the reflectance as a function of the angle of reflection. The experimental setup is shown in Figure 2. The halogen lamp served as the light source. After reflecting from the sample surface at an angle of incidence *Φ*, the reflected light was collected by the OceanOptics-HR2000+ spectrometer within the range of 350 nm to 1050 nm.

The prepared samples were structurally characterized using a scanning electron microscope (SEM, FEI Quanata FEG 250—FEI Company, Hillsboro, OR, USA). A qualitative analysis was performed using a commercial micro-Raman MonoVistaUV-vis confocal Raman Microscope (S&I Spectroscopy & Imaging GmbH, Warstein, Germany) equipped with a He-Cd laser emitting at 325 nm and an Ar^+^ laser emitting at 514 nm. Raman spectra were collected at room temperature in a backscattering geometry with a spectral resolution of approximately 1 cm^−1^.

## 3. Results and Discussion

**Figure 3:**
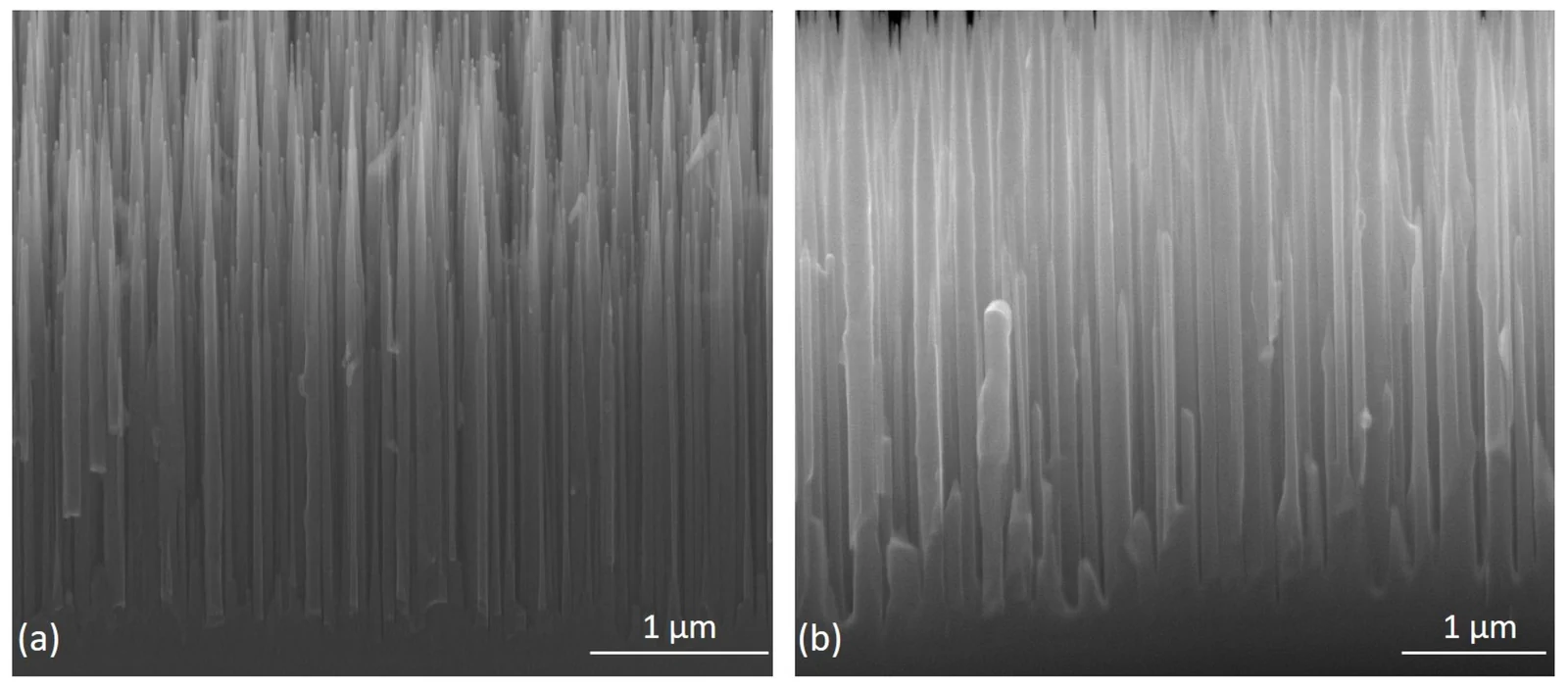
Cross-sectional SEM images of GaP nanowires before and after PtSe_2_ growth. (**a**) As-grown GaP nanowire array on GaP(111)B substrate. (**b**) GaP nanowires after deposition of a thin Pt layer and subsequent selenization, forming a continuous PtSe_2_-based film on the nanowire sidewalls and on the exposed GaP surface.

Three types of substrates were used for the fabrication of PtSe_2_ layers: GaP substrate, nanostructured GaP substrate, and c-plane sapphire substrate. While flat GaP and sapphire substrates are monocrystalline with a roughness below 0.2 nm, nanostructured GaP has a rough surface. A high-density GaP nanowire array grown by MOVPE is shown in Figure 3a. The nanowire heights range from 1.5 to 2.5 μm. Cross-sectional SEM micrographs further illustrate how the selenization step modifies the morphology of the GaP nanowire arrays. While the as-grown nanowires (Figure 3a) exhibit smooth, well-defined facets and a sharp interface with the planar GaP substrate, after Pt deposition and selenization (Figure 3b) the entire nanowire array is covered by a continuous PtSe_2_-based film. The sidewalls of the nanowires become slightly rounded and the apparent diameter of the pillars increases. The dense, vertically oriented nanowire ensemble and the conformal coating provide a large effective interfacial area, which is expected to enhance light absorption and charge collection in the PtSe_2_/GaP-based photoactive devices.

**Figure 4:**
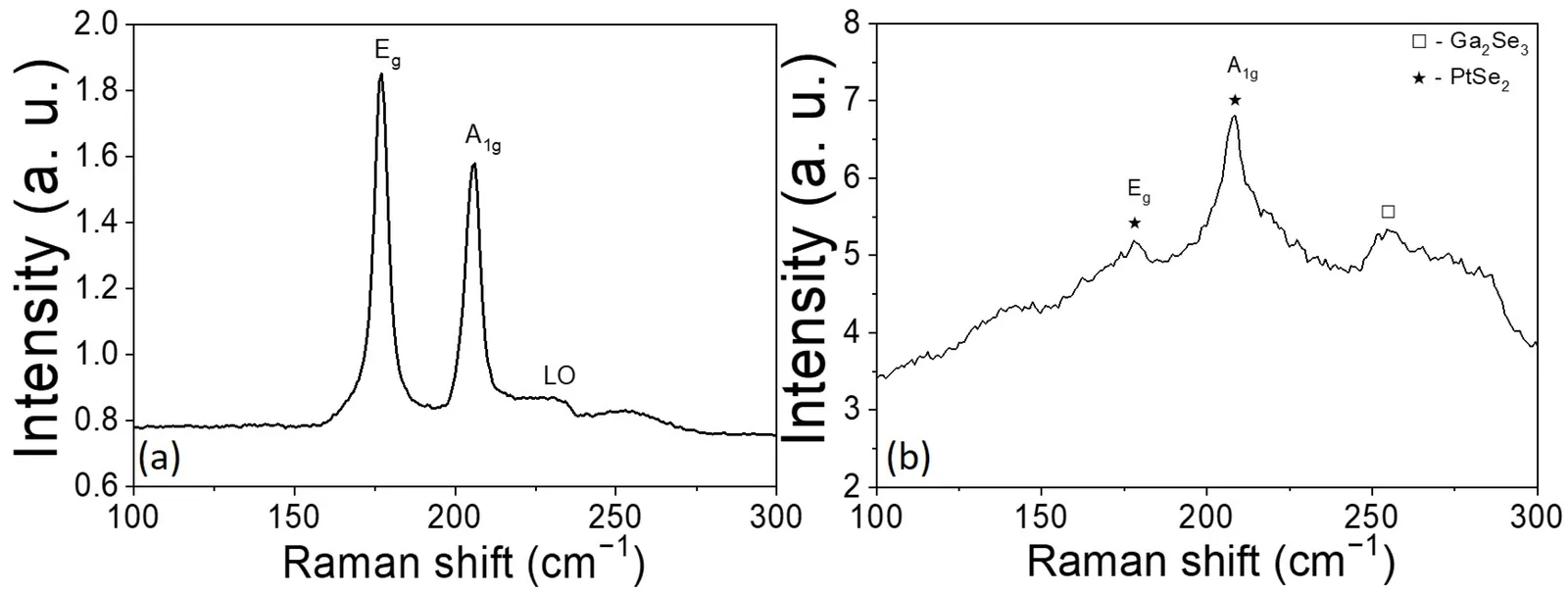
Raman spectra of PtSe_2_ film prepared on (**a**) c-plane sapphire substrate and (**b**) GaP substrate.

After selenization annealing, the growth of PtSe_2_ was confirmed by Raman spectroscopy (Figure 4). However, Ga_2_Se_3_ was also detected in the case of GaP substrates. We assume that, at the initial stage of annealing, phosphorus diffuses from the GaP substrate toward the Pt layer, as evidenced by its presence in both the interfacial amorphous layer and the Pt-rich region. While a limited initial interaction of selenium with the GaP surface cannot be excluded, potentially facilitated by the discontinuous character of the Pt layer, this effect is likely transient and overshadowed by the dominant outward diffusion of P. This indicates that the selenization process may start simultaneously at both the GaP and Pt layers. As the selenization continues, Se atoms cannot reach the GaP surface because PtSe_2_ grows rapidly, so not all of the GaP substrate is converted to Ga_2_Se_3_. Our experiments demonstrate that a 3 nm thick Pt layer effectively prevents Se from reaching the GaP surface, inhibiting the formation of a continuous Ga_2_Se_3_ interlayer between the GaP substrate and the PtSe_2_ layer. Ga_2_Se_3_ is a semiconductor that exhibits n-type conductivity with a bandgap of 1.85 eV [29,30].

Raman spectroscopy measurements were performed on as-synthesized PtSe_2_ thin films deposited on GaP substrates, both with and without nanowires, and on the c-plane sapphire substrate. The Raman spectra of the PtSe_2_ on the c-plane sapphire (Figure 4a) exhibit the E_g_ mode at around 177 cm^−1^ and the A_1g_ mode at 206 cm^−1^, confirming the growth of the 1T phase. In addition to the E_g_ and A_1g_ peaks, a small peak at 230 cm^−1^, attributed to a longitudinal optical (LO) mode, was observed. The presence of PtSe_2_ on the GaP substrate is confirmed in Figure 4b. The Raman spectrum exhibits characteristic peaks corresponding to the E_g_ (~178 cm^−1^) and A_1g_ (~208 cm^−1^) vibrational modes of the deposited PtSe_2_ thin film, along with a weak peak attributed to Ga_2_Se_3_ at around 254 cm^−1^.

**Figure 5:**
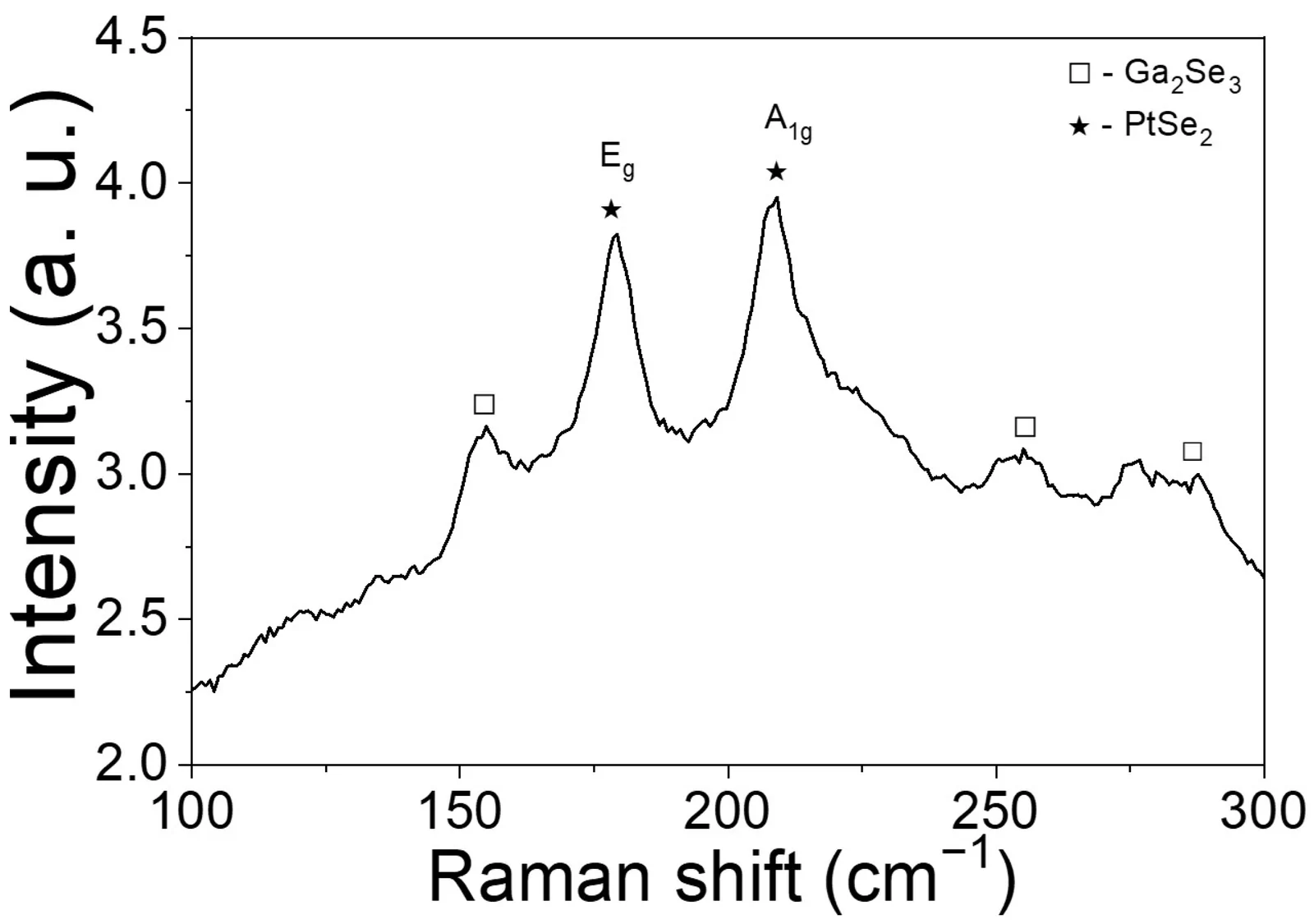
Raman spectra of GaP NWs/PtSe_2_ structure prepared by selenization at 550 °C for 30 min with the Ga_2_Se_3_ peaks.

Raman spectra of GaP NWs substrate with PtSe_2_ thin film are demonstrated in Figure 5. Aside from the expected PtSe_2_ Raman modes, several additional peaks related to Ga-Se phases, consistent with Ga_2_Se_3_, were observed. For PtSe_2_, two key vibrational modes appear at about 180 and 208.5 cm^−1^, with a weaker signal near 230 cm^−1^. Comparing these peaks with phonon frequency predictions for perfect crystals, the ones at 180 and 208.5 cm^−1^ are assigned to the E_g_ and A_1g_ modes, respectively. The E_g_ mode arises from in-plane vibrations of Se atoms, while the A_1g_ mode corresponds to out-of-plane vibrations. The Raman peaks associated with Ga_2_Se_3_ were observed at around 155, 250, and 290 cm^−1^. As mentioned in Ref [2], the peaks at 250 cm^−1^ and 290 cm^−1^ are due to short-range interactions within the defect zincblende unit cell. In contrast, the lower-frequency peak is associated with long-range ordering in the monoclinic superstructure of β-Ga_2_Se_3_ [31].

While Raman spectra clearly indicate the formation of Ga-Se related phases at the GaP interface, they do not by themselves distinguish between pure Ga_2_Se_3_ and more complex Ga-O-Se mixtures. Cross-sectional TEM and STEM-EDS analysis indicate that Ga_2_Se_3_ is present as crystalline inclusions within the granular Pt-rich layer, rather than forming a continuous interfacial layer, while the region near the substrate remains partially amorphous and contains a mixed Ga-O-P composition.

**Figure 6:**
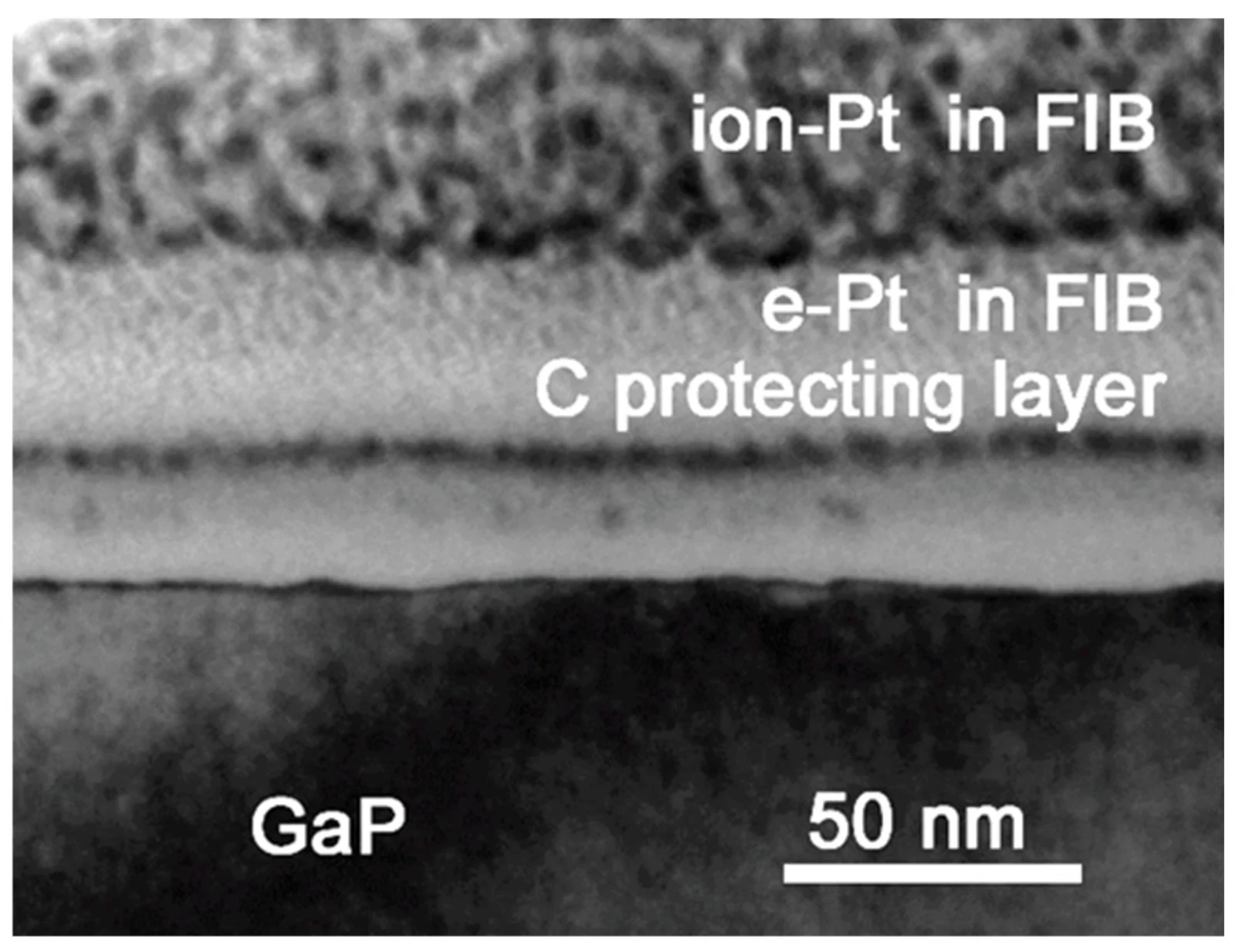
Cross-section conventional TEM micrograph of selenized Pt layer on the top of GaP substrate and protecting layers after a final thinning.

Cross-sectional conventional TEM on selenized Pt layers grown on flat GaP substrates reveals a multi-layered structure consisting of the GaP substrate, a relatively thick interfacial layer, and a Pt-rich top layer, together with the protective capping layers used for TEM sample preparation (Figure 6). The large separation between the GaP surface and the Pt-rich layer can be explained by the presence of a pre-existing native oxide, whose thickness increased during the high-temperature selenization step.

**Figure 7:**
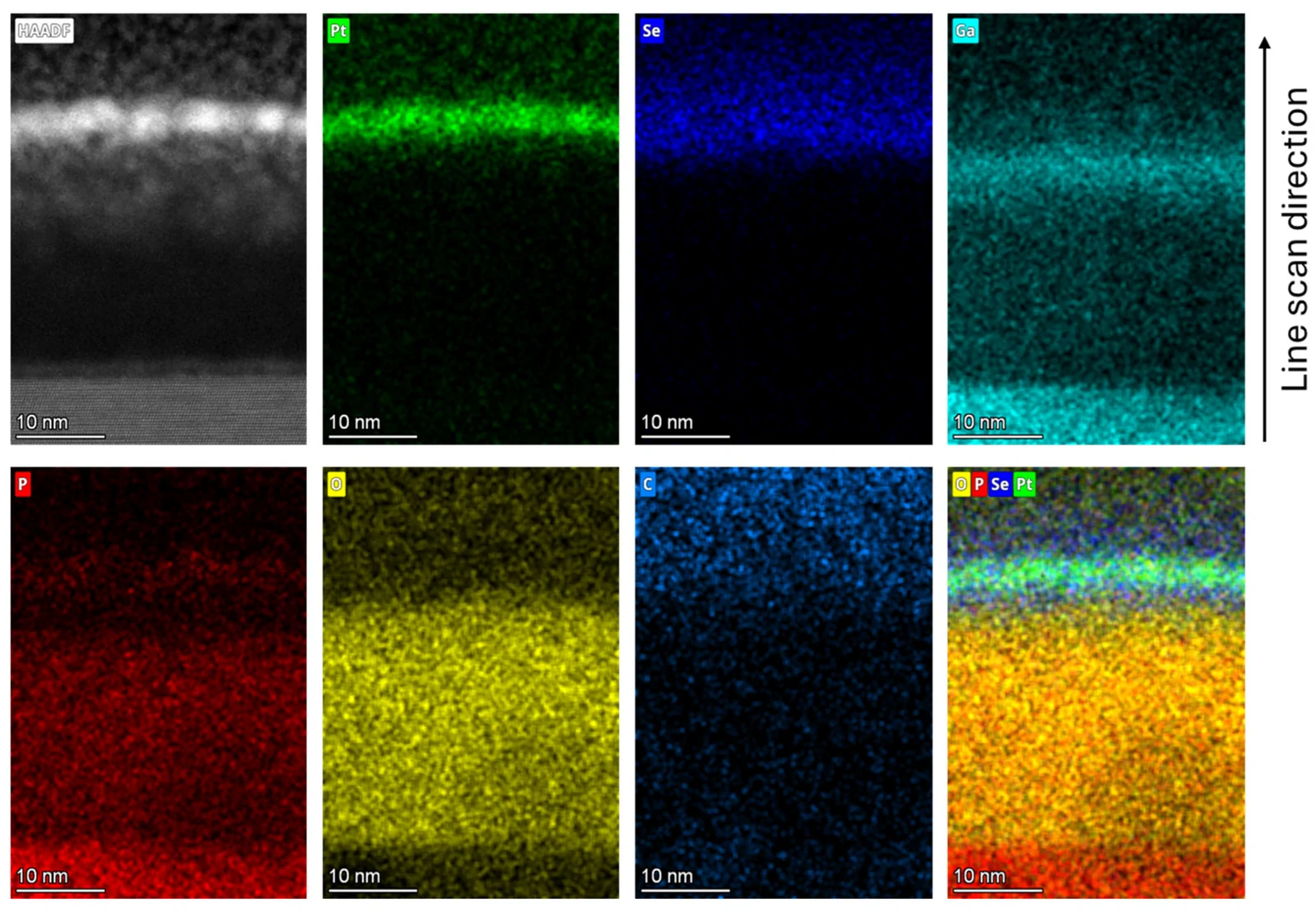
STEM-EDS elemental maps and the corresponding HAADF micrograph of the selenized Pt layer on the top of the GaP substrate.

**Figure 8:**
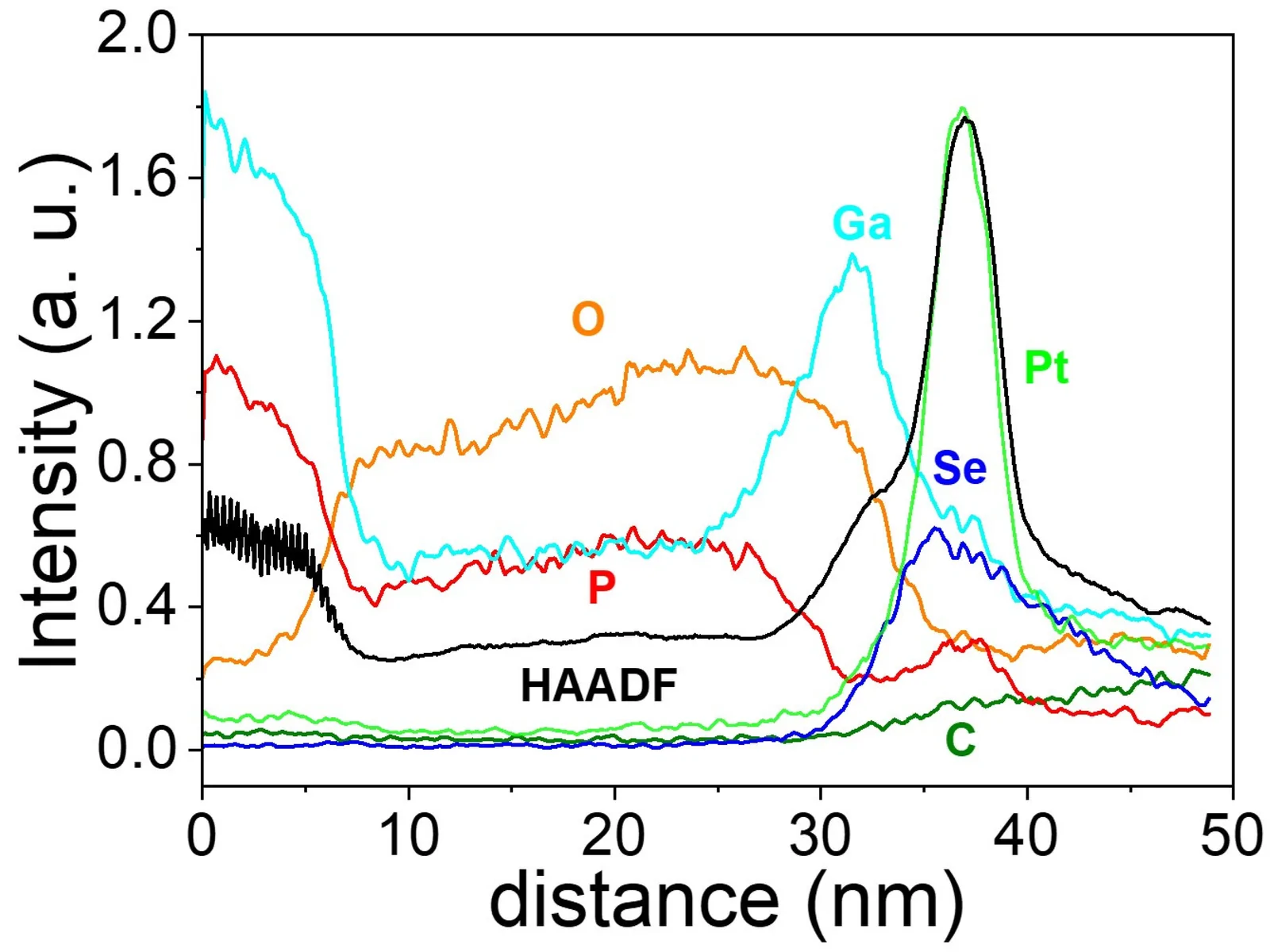
EDS signal line-scans calculated from EDS mapping shown in Figure 7 in the arrow direction.

STEM-EDS mapping (Figure 7) and the corresponding integrated line scans (Figure 8) show pronounced redistribution of elements across the heterostructure during selenization, with Ga extending upward from the GaP substrate and Se penetrating into the Pt-rich top region. Although the TEM lamella was prepared using a Ga ion focused beam and a minor contribution of implanted Ga cannot be excluded, the elemental maps and contrast variations in the cross-sectional images indicate that most of the Ga in the upper part of the structure originates from diffusion out of the GaP substrate and its reaction with the Pt-rich layer. The interfacial region between GaP and the Pt-rich layer exhibits strong Ga and O signals and a largely diffuse HAADF contrast, consistent with an amorphous Ga–O-rich interlayer formed from native or adsorbed oxygen on the GaP surface prior to Pt deposition. Within the Pt-rich layer, the O signal is strongly attenuated, most likely due to absorption of low-energy X-rays in the Pt-rich matrix, whereas Se is largely consumed inside the Pt-rich layer and its immediate surroundings during selenization.

**Figure 9:**
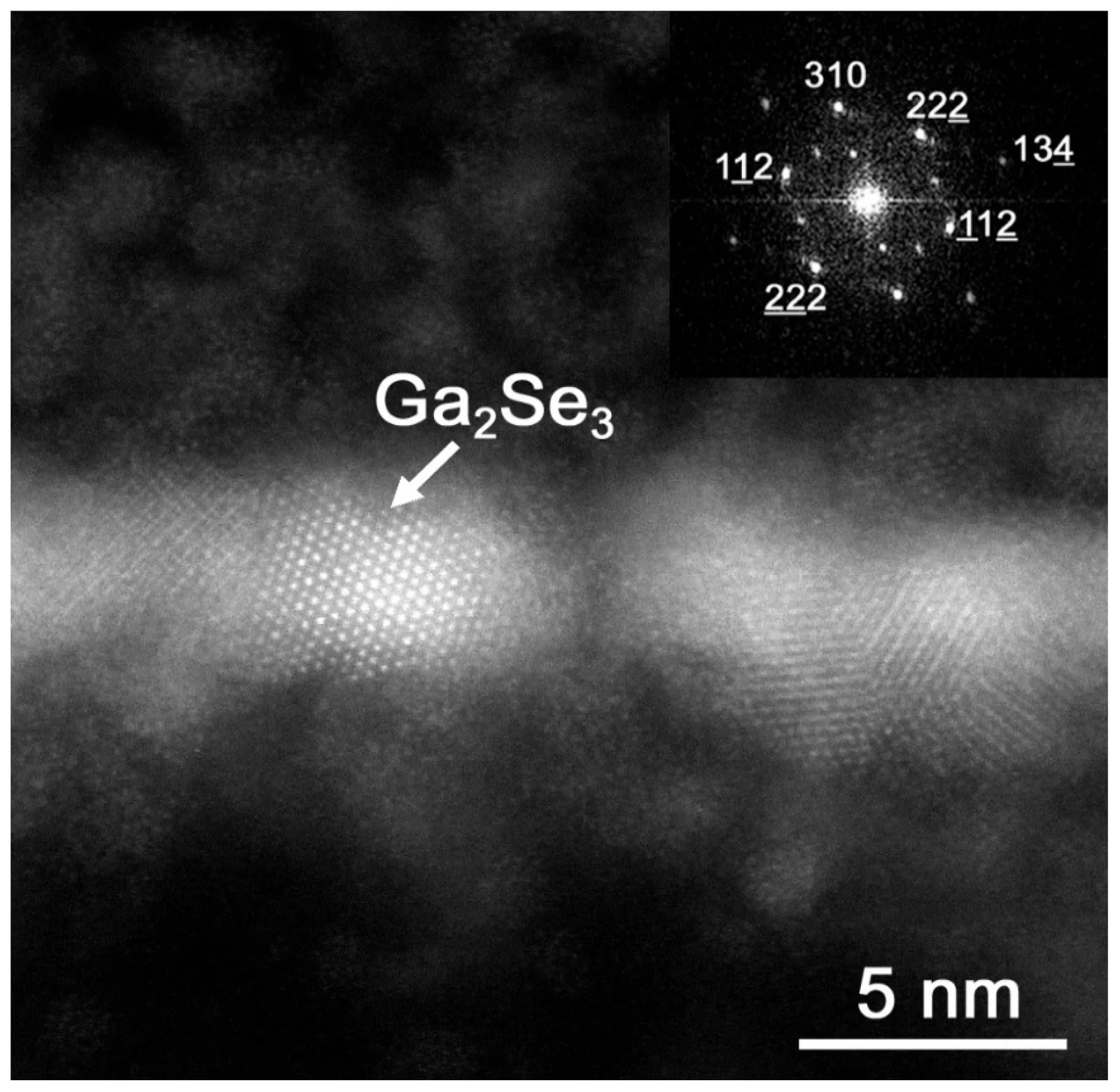
High-resolution HAADF-STEM image of the PtSe_2_-rich region and the underlying interfacial region, showing discrete α-Ga_2_Se_3_ crystallites embedded in the nanocrystalline PtSe_2_-rich region and an adjacent predominantly amorphous Ga_2_O_3_-rich interlayer.

The high-resolution HAADF-STEM micrograph in Figure 9 resolves the local crystallinity within the interfacial region. Well-defined lattice fringes appear only in small crystallites that can be indexed to monoclinic Ga_2_Se_3_, indicating that it forms nanocrystalline grains with typical sizes of a few nanometers at the interface. The adjacent Ga–O-rich band shows mainly smooth, featureless contrast without clear lattice planes, which is characteristic of an amorphous layer. The Pt-rich region above the interface exhibits fine-grained contrast consistent with nanocrystalline PtSe_2_, but no larger, well-ordered grains are observed in this cross-section. Therefore, Ga_2_Se_3_ does not form a continuous interfacial layer but occurs as discrete crystallites within the PtSe_2_-rich region.

Overall, the structural characterization shows that selenization of thin Pt films on GaP produces a multilayer heterostructure in which a nanocrystalline PtSe_2_-rich top layer, containing embedded Ga_2_Se_3_, is separated from the GaP substrate by a relatively thick, predominantly amorphous Ga–O–P interfacial region. Cross-section TEM, STEM-EDS and high-resolution HAADF-STEM demonstrate strong redistribution of Ga and Se during selenization, localization of Ga and O in the amorphous interlayer, and the presence of only small monoclinic Ga_2_Se_3_ crystallites within the Pt-rich layer rather than a continuous Ga_2_Se_3_ film. This chemical composition was further confirmed by XPS analysis.

**Figure 10:**
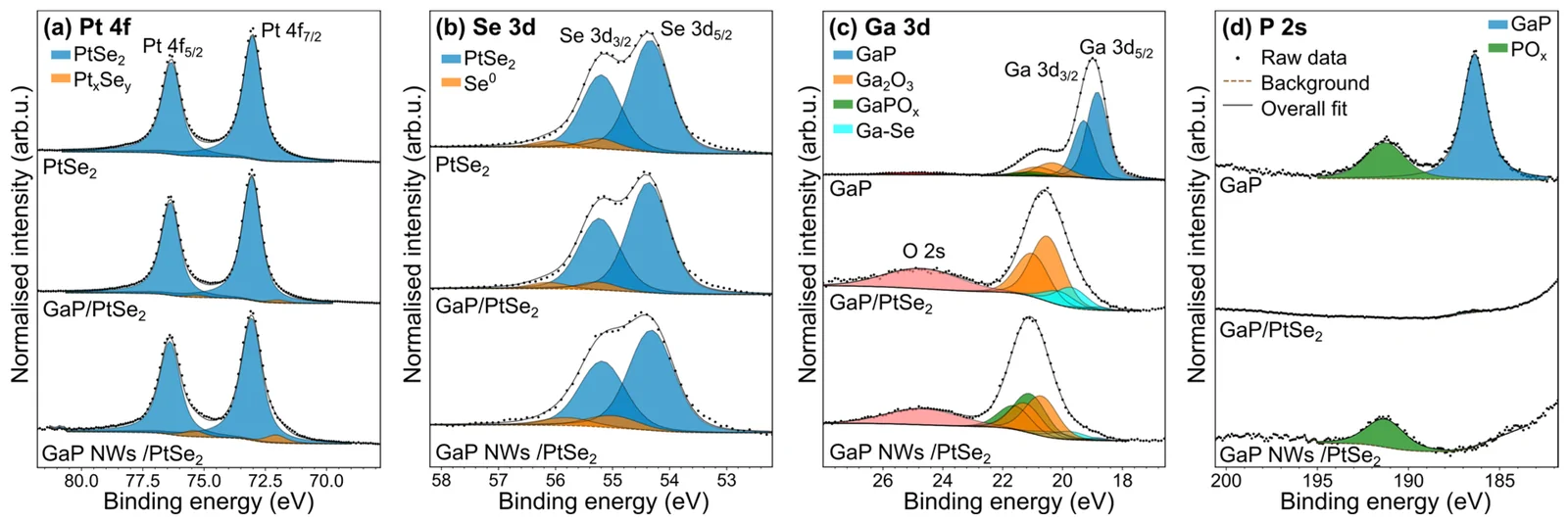
XPS spectra of (**a**) Pt 4f, (**b**) Se 3d, (**c**) Ga 3d, and (**d**) P 2s for the GaP NWs/PtSe_2_ and planar GaP/PtSe_2_ heterostructures. XPS spectra of a PtSe_2_ reference film grown on c-plane (0001) sapphire substrate are included for comparison. The spectra are normalised to their maximum intensities.

Figure 10 compares the XPS spectra of the GaP NWs/PtSe_2_ and GaP/PtSe_2_ heterostructures with those of the GaP and PtSe_2_ reference samples. The Pt 4f spectra of both heterostructures display a dominant doublet with the Pt 4f_7/2_ at approximately 73.0 eV (Figure 10a), characteristic of Pt bonded to Se in PtSe_2_, accompanied by a lower-binding-energy shoulder near 72.0 eV that can be assigned to Pt-Se environments with slightly different stoichiometry [12]. No metallic Pt contribution is observed within the detection limit, indicating that the deposited Pt was completely converted into Pt-Se phases under the selenization conditions used. This is consistent with the nanocrystalline PtSe_2_-rich top layer observed by STEM-EDS.

The Se 3d spectra (Figure 10b) consist of a main doublet with the Se 3d_5/2_ peak at 54.4 eV, assigned to the characteristic of Se in PtSe_2_, together with a higher-binding-energy component at 55.2 eV attributed to elemental Se [10,12,32]. The Se/Pt atomic concentration ratios, derived from the Se 3d and Pt 4f peak areas, are close to 2 for all samples and slightly exceed the stoichiometric value for the GaP/PtSe_2_ heterostructures.

Figure 10c and d show the Ga 3d and P 2s XPS spectra of the GaP(111)B wafer and the GaP/PtSe_2_ heterostructures. The P 2p spectra were also measured, but are omitted here due to their overlap with a Se Auger signal. For the GaP wafer, the Ga 3d and P 2s spectra are dominated by the GaP components, with the Ga 3d_5/2_ and P 2s peaks located at 18.8 and 186.4 eV, respectively. The Ga 3d spectrum is accompanied by weaker doublets with Ga 3d_5/2_ binding energies of 20.3 and 21.0 eV, assigned to Ga_2_O_3_ and gallium phosphates species with different stoichiometries (GaPO_x_, 1 < x ≤ 4), respectively [33,34]. The P 2s spectrum likewise exhibits a weaker higher-binding-energy component at 191.2 eV, corresponding to the component at 133.7 eV observed in the 2p spectrum, which is attributed to surface phosphate species (PO_x_) [33,35] associated with the native oxide layer.

The Ga 3d and P 2s spectra of both GaP/PtSe_2_ heterostructures are dominated by the Ga_2_O_3_ and GaPO_x_ components, while the GaP signal is strongly suppressed. In addition, a component with a Ga 3d5/2 binding energy of 19.6 eV is observed. Binding energies in the range of 19.2–19.8 eV have been reported for Ga selenides [36,37,38], consistent with the presence of Ga–Se and Ga_2_Se_3_ species observed by Raman, TEM, and STEM-EDS. This assignment is further supported by the slightly higher Se content observed in the Se 3d spectra than expected for stoichiometric PtSe_2_. The close proximity of the reported Se 3d5/2 binding energies of Ga selenides (54.1–54.6 eV) to that of PtSe_2_ (54.4 eV) makes it difficult to distinguish their respective contributions in the Se 3d spectra.

Overall, the XPS analysis confirms that the near-surface region of PtSe_2_ films grown on both planar and nanostructured GaP substrates consists of a PtSe_2_-rich layer with a minor contribution from gallium selenides, in contact with a gallium-oxide-dominated interfacial region formed on GaP, and containing additional Ga-O-P oxide species These findings are consistent with a heterogeneous PtSe_2_-rich/Ga_2_O_3_-rich/GaP interface containing discrete Ga_2_Se_3_ crystallites, as derived from the cross-sectional TEM and STEM-EDS analyses.

**Figure 11:**
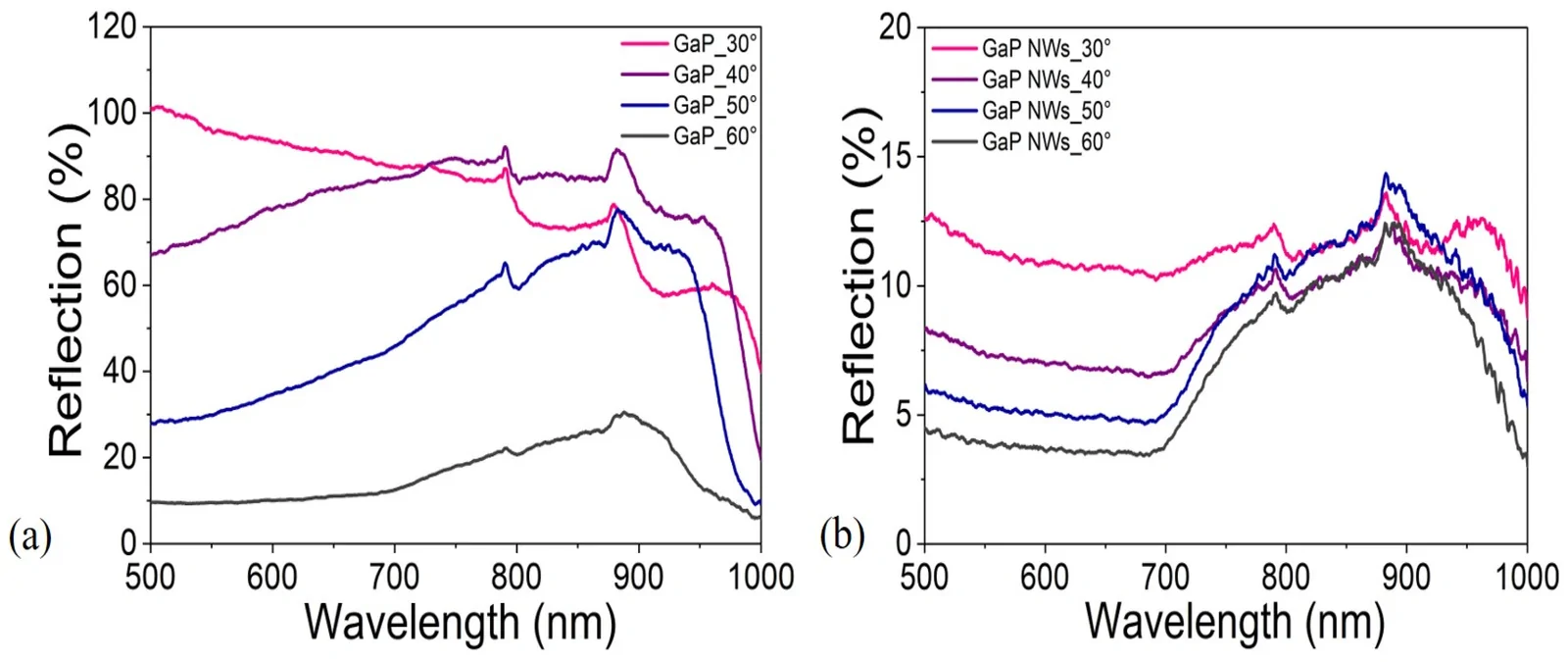
Reflectance of PtSe_2_ thin film deposited on (**a**) GaP substrate and (**b**) GaP NW substrate measured under four different angles of incidence, *Φ*: 30°, 40°, 50°, and 60°.

The reflectance measurements were examined from the point of view of (i) different angles of incidence, *Φ*, and (ii) different substrates. The peaks in the reflectance spectra corresponding to the absorbance of the PtSe_2_ thin film do not shift with either parameter (i) or (ii). The reflectance spectra of PtSe_2_ deposited on a GaP substrate (Figure 11a), as well as on a GaP NW substrate (Figure 11b) are shown. The reflectance spectra were measured at four angles of incidence (*Φ*): 30°, 40°, 50°, and 60°. At approximately 950 nm, the local maxima shift with angle of incidence for both substrates, and thus, they do not correspond to material absorbance. Nevertheless, for both substrates, several local maxima retain their spectral positions with respect to the angle of incidence: 750 nm (1.65 eV), 790 nm (1.57 eV), and 880 nm (1.41 eV).

**Figure 12:**
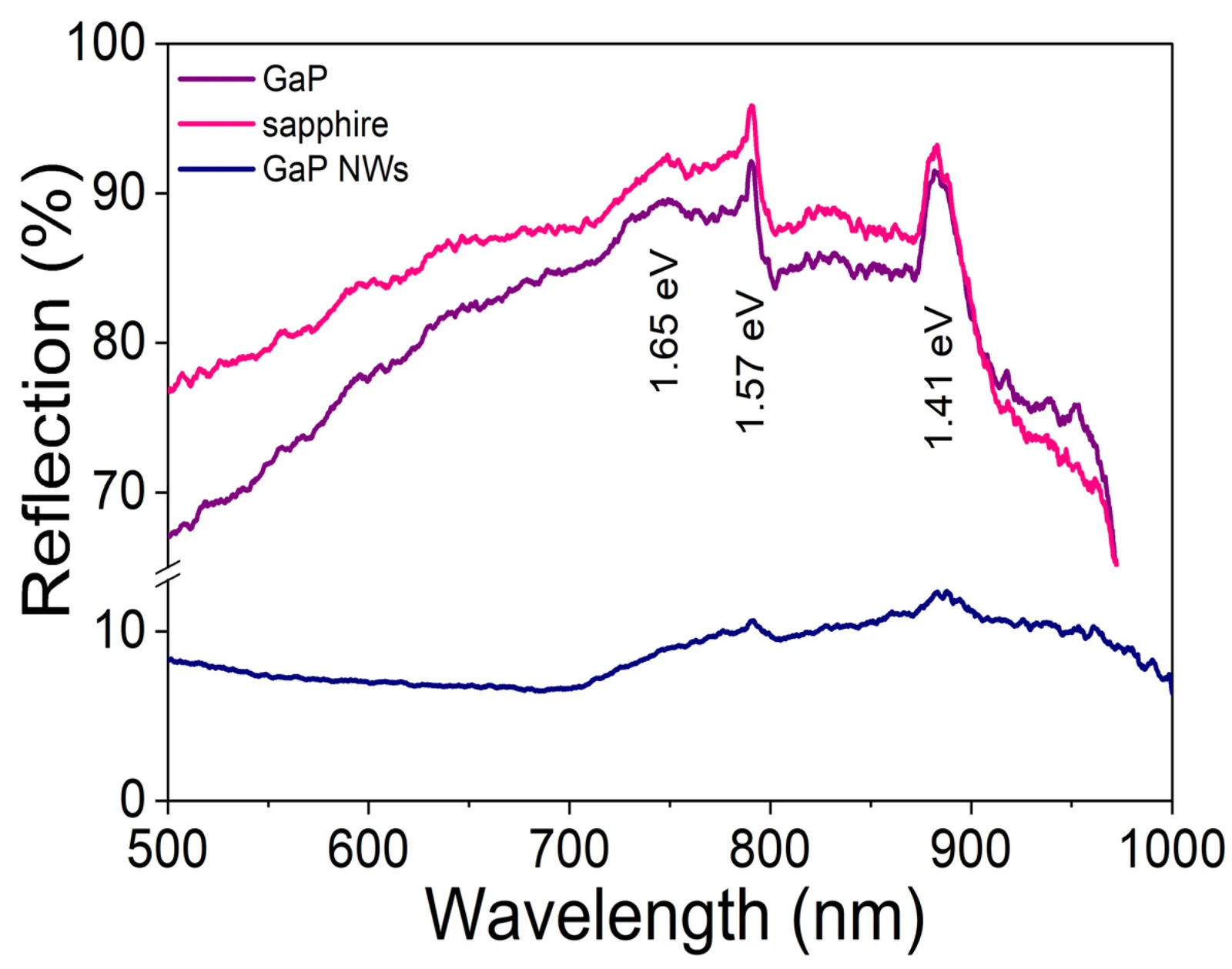
Reflectance for PtSe_2_ deposited on three different substrates: GaP, c-plane sapphire, and GaP NW. The angle of reflection is 40°.

For detailed analysis, we compared reflectance at 40° for three substrates: c-plane sapphire, GaP, and GaP NW substrate (Figure 12). The graph shows a decrease in reflectance for the GaP NW substrate due to its nanostructured nature. As observed, all measured spectra exhibit similar characteristics regardless of the substrate. Therefore, we can conclude that all reflectance maxima are related to PtSe_2_.

Published data demonstrate that the 1L PtSe_2_ is an indirect semiconductor exhibiting a band gap between 1.2 and 1.8 eV [19,39,40]. The bandgap energy decreases dramatically with increasing PtSe_2_ layer thickness, transiting the material to become a semi-metal. For 2L of PtSe_2_, the bandgap energy is approximately 0.5 eV [40]. However, a recent publication shows that optical absorption of 1L and few-layered PtSe_2_ arises from direct transitions also at wavevectors k ≠ 0, which leads to higher energy transitions for few-layered PtSe_2_ [41].

In Figure 12, three visible peaks are detected that correspond to band gap energies of 1.65 eV, 1.57 eV, and 1.41 eV. Measured absorption maxima are consistent with thin semiconducting PtSe_2_ layers on all measured substrates and with previously reported band-gap values for 1L and few-layer PtSe_2_ [19,39,40,41].

**Figure 13:**
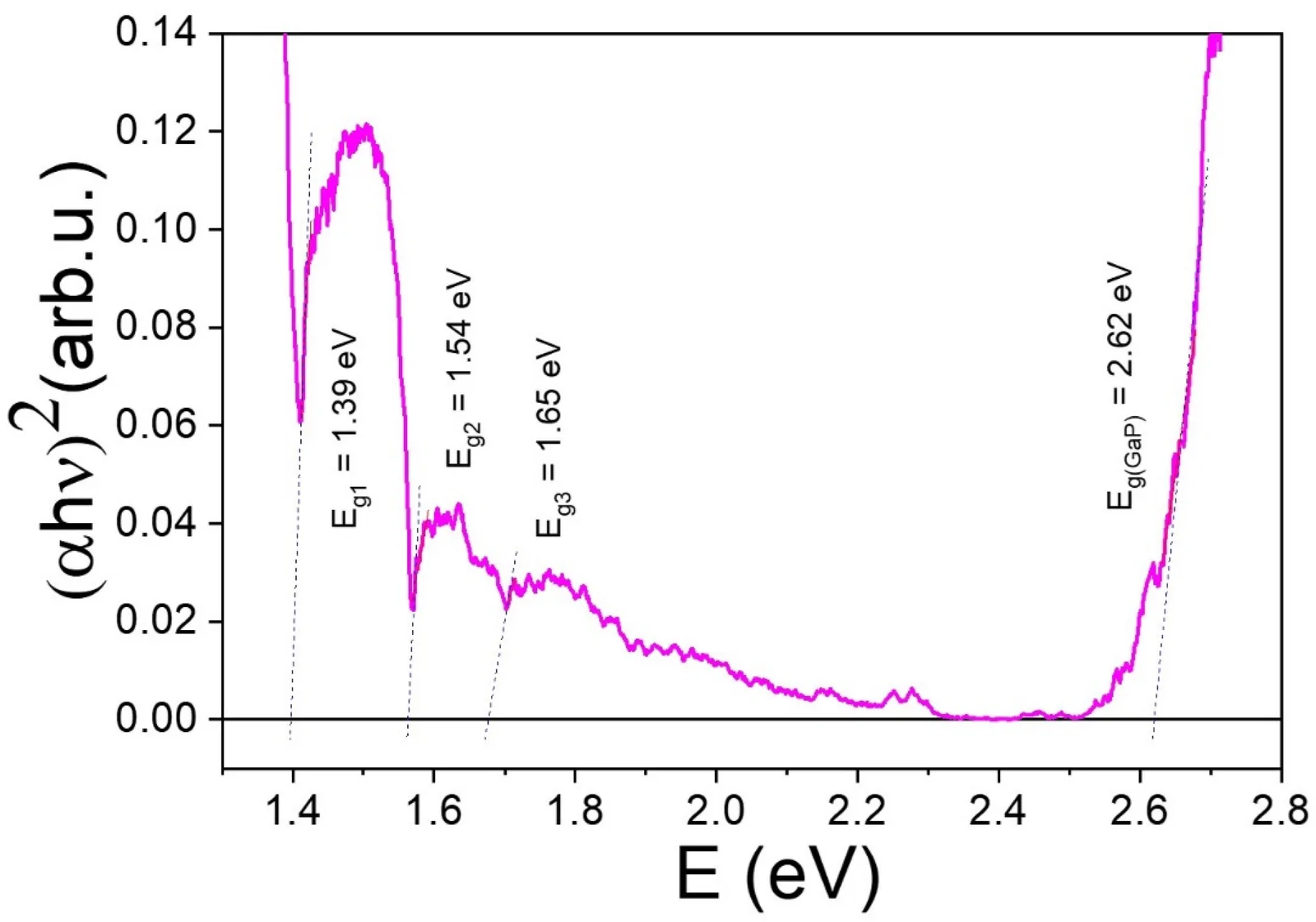
Modified absorbance characteristic of PtSe_2_ on GaP substrate for the use of Tauc’s formula.

The Tauc’s method is widely used due to its simplicity and effectiveness in estimating band gap values for crystalline, polycrystalline, and amorphous semiconductors. However, accurate band gap determination depends on proper selection of the transition type and careful identification of the linear region in the Tauc’s plot. Figure 13 presents the modified absorbance for PtSe_2_ on GaP substrate, which represents the Tauc’s transformation for optical band-gap evaluation.

The bandgap energy of approximately 2.62 eV corresponds to the bulk GaP material. Furthermore, we determined three absorption edges corresponding to the bandgap energies 1.39 eV, 1.54 eV, and 1.65 eV, which can be related to optical transitions in thin PtSe_2_ and are in agreement with published data for 1L and few-layered PtSe_2_ [19,39,40,41]. The observed reflectance maxima in the 1.4–1.7 eV range and the corresponding Tauc-derived absorption edges are far below the reported bandgap energies of GaP, Ga_2_Se_3_ and Ga_2_O_3_, supporting their assignment to 1L or few-layered PtSe_2_-related transitions.

Van der Pauw–Hall measurements of the PtSe_2_ reference film on sapphire revealed p-type conduction, with a Hall hole concentration of *p* = 1.4 × 10^21^ cm^−3^ and a Hall mobility of 21 cm^2^ V^−1^ s^−1^. The high effective hole concentration indicates degenerate, semimetal-like transport. Nevertheless, the measured mobility is within the experimentally relevant range for polycrystalline PtSe_2_ films prepared by thermally assisted conversion (TAC) of pre-deposited Pt layers. Hall mobilities of 5–13 cm^2^ V^−1^ s^−1^ [42] have been reported for TAC-grown PtSe_2_ films, whereas nanocrystalline TAC-grown PtSe_2_ films exhibited effective Hall mobilities of 2.1–15.3 cm^2^ V^−1^ s^−1^ [43]. The mobility of TAC-grown films is affected by grain boundaries, crystallite orientation, structural disorder, charged defects, deviations from ideal Se:Pt stoichiometry, and interface scattering. These scattering mechanisms can coexist with a high carrier density; therefore, the observed Hall concentration and mobility are physically compatible. The reported volume carrier concentration was obtained by converting the measured Hall sheet density using the effective PtSe_2_ film thickness.

**Figure 14:**
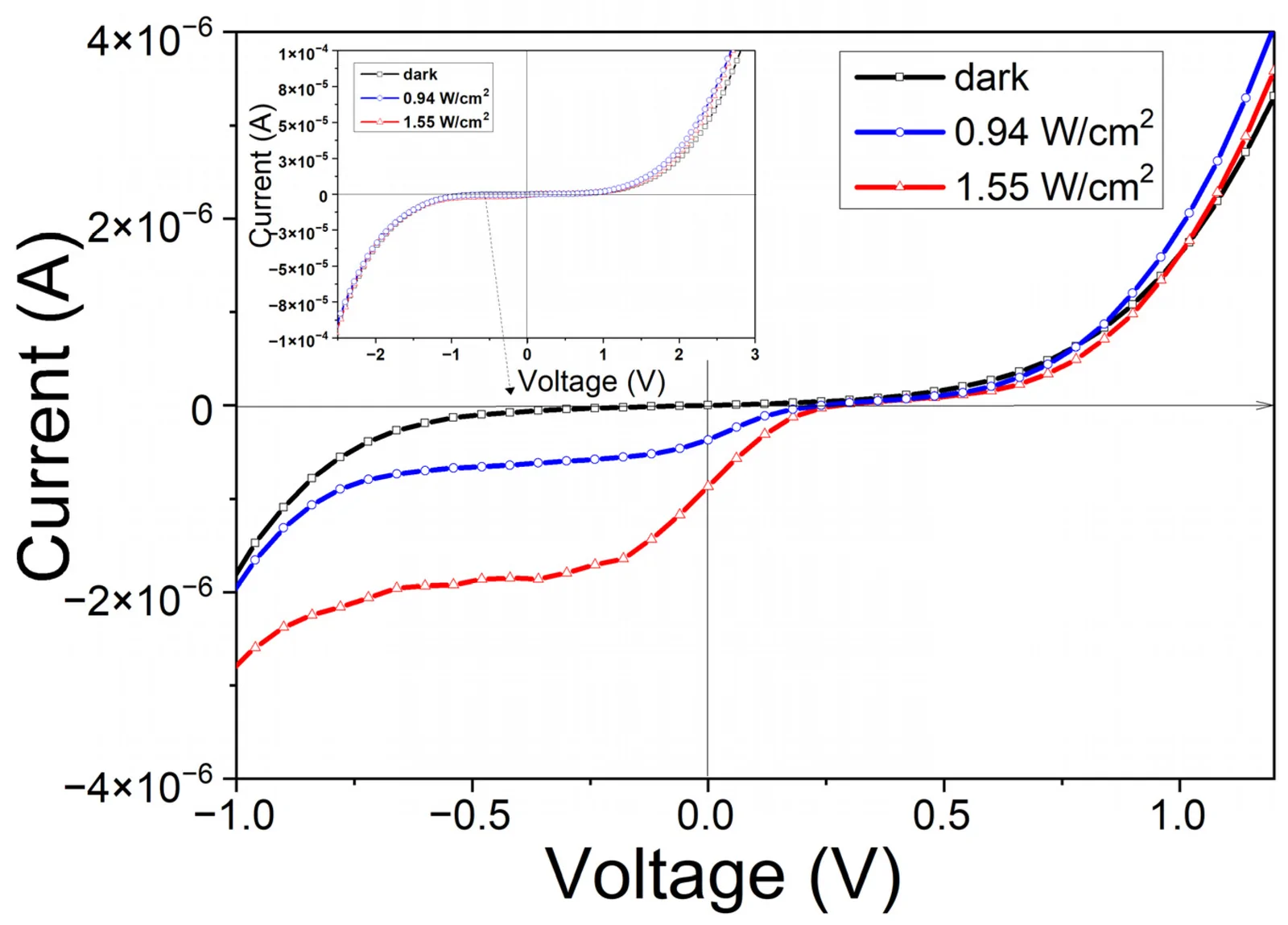
*I*-*V* characteristics of PtSe_2_-rich/Ga_2_O_3_-rich/GaP heterostructure containing Ga_2_Se_3_ crystallites at various illumination levels. (Inset: Extended *I*-*V* characteristic for supply voltage −2.5 to 3V).

Two-terminal current–voltage characteristics measured between the backside indium contact on p-GaP and the top indium contact on PtSe_2_ exhibit rectifying behavior consistent with a heterojunction interface in region −1 V, 1 V as shown in Figure 14. Under white-light illumination the reverse current shows a considerable photocurrent contribution, which can be qualitatively interpreted within a p-n-like junction framework involving the GaP substrate and the PtSe_2_-based top layer. For reverse voltages higher than −1 V, the reverse current increases sharply and can be attributed to tunnelling through the thin barrier probably caused by the presence of a Ga_2_O_3_-rich thin layer at the interface as seen in the inset of Figure 14. In the forward direction, the knee voltage can be identified in the dark in the vicinity of 0.5 V. Under illumination, the opening voltage for the photodiode is around 0.25 V and the generated photocurrent shows a significant increase and saturation in the range of −0.2 to −1 V at different illumination intensities.

**Figure 15:**
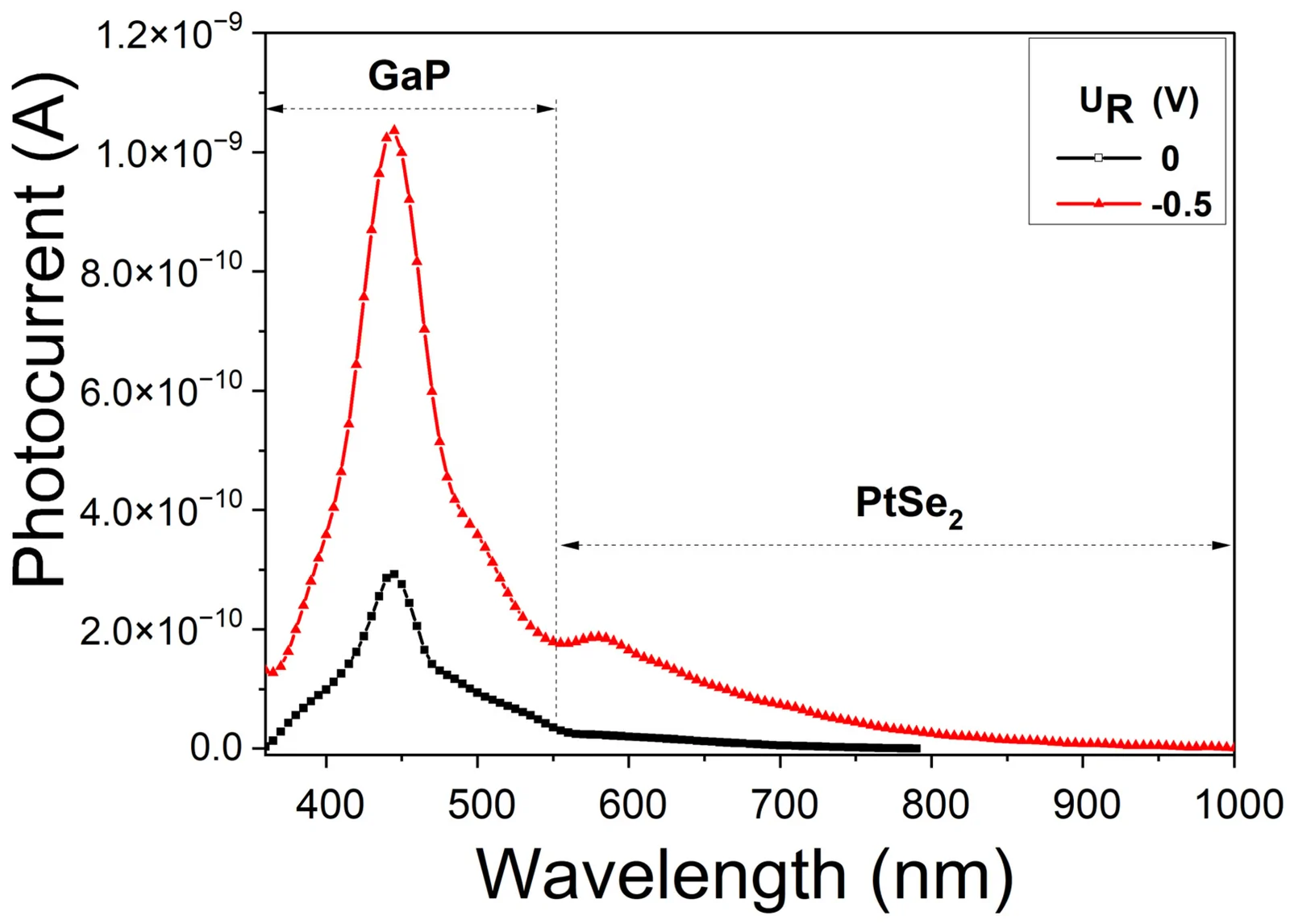
Photocurrent generation in PtSe_2_-rich/Ga_2_O_3_-rich/GaP heterostructure containing Ga_2_Se_3_ crystallites in the wavelength range 350–1000 nm at 0 V and reverse voltage −0.5 V.

Spectral photocurrent between 350 and 1000 nm was measured on the previously analyzed photodiode (Figure 15) at 0 V and reverse voltage −0.5 V. The measured spectral characteristics can be divided into two regions: a shorter-wavelength mode (350–550 nm) dominated by carrier generation in GaP, and a longer-wavelength mode (550–1000 nm) attributed to absorption in PtSe_2_. These data demonstrate the broadband photoresponse of the PtSe_2_-rich/Ga_2_O_3_-rich/GaP heterostructure containing Ga_2_Se_3_ crystallites and correspond to the structural complexity as revealed by TEM measurements.

## 4. Conclusions

In summary, we have demonstrated the growth of PtSe_2_ films on flat and nanostructured GaP substrates by thermally assisted selenization of thin Pt layers, and we have investigated their structural, optical and electrical properties. Raman and optical measurements confirm the presence of PtSe_2_ and Ga-Se-related phases, while cross-sectional TEM, STEM-EDS and XPS reveal a heterogeneous interface comprising a PtSe_2_-rich top region containing discrete Ga_2_Se_3_ crystallites and an underlying predominantly amorphous Ga_2_O_3_-rich interlayer on GaP.

Two-terminal electrical and photoelectrical measurements show rectifying characteristics and broadband photoresponse, with contributions from both the GaP substrate and the PtSe_2_ layer, indicative of an effective heterojunction interface. However, the structural complexity of the interfacial layer show that the device is more conservatively described as a PtSe_2_-rich/Ga_2_O_3_-rich/GaP photodiode-like heterostructure containing Ga_2_Se_3_ crystallites.

These findings highlight both the promise and the challenges of integrating PtSe_2_ with III-V substrates: while interdiffusion and oxide formation can yield functional photoresponsive interfaces, they also complicate band-structure engineering and device reproducibility. Controlling the chemistry and microstructure of the interfacial layer will be essential for realizing well-defined PtSe_2_/III-V heterojunctions suitable for advanced optoelectronic and transistor applications.

## Data Availability

The original contributions presented in this study are included in the article. Further inquiries can be directed to the corresponding author.
